# Social construction of online sex work: a social welfare analysis of MiChat-mediated sexual labor in Jayapura, Indonesia

**DOI:** 10.3389/fsoc.2026.1756800

**Published:** 2026-04-24

**Authors:** Fitrine Christiane Abidjulu, Amos Kesi Shibuki Marini, Usman Idris

**Affiliations:** 1 Social Welfare Studies Program, Cenderawasih University, Jayapura, Indonesia; 2 Department of Anthropology, Cenderawasih University, Jayapura, Indonesia

**Keywords:** MiChat, multidimensional wellbeing, online sex work, social construction, structural inequality

## Abstract

This study aims to analyze how online sex work practices through the MiChat application in Jayapura City are socially constructed and related to multidimensional wellbeing. The study employed a qualitative approach with in-depth interviews with three informants purposively selected based on varying socioeconomic backgrounds. Data analysis was conducted thematically, drawing on Berger and Luckmann's social construction theory, encompassing the processes of externalization, objectivation, and internalization. The results indicate that online sex work is not a homogeneous phenomenon, but rather a practice negotiated within the context of structural constraints, such as economic pressure, social stigma, and weak institutional support. This practice serves as a short-term livelihood strategy, but simultaneously reproduces vulnerabilities in economic, social, psychological, spiritual, and health dimensions. Furthermore, the findings suggest that individuals involved are more accurately understood as victim-survivors actively navigating choices within unequal conditions. This study contributes by integrating social construction and multidimensional wellbeing perspectives to understand digital-based informal work in peripheral urban contexts. Policy implications emphasize the importance of inclusive and gender-sensitive structural-based interventions.

## 1 Introduction

Online sex work has become a social phenomenon that is increasingly prominent amid the advancement of the digital age. Along with the rapid development of information and communication technology, various new opportunities have emerged that are not only positive but also open up new spaces for digitally mediated informal labor practices, including online sex work, which are often socially stigmatized within specific cultural contexts (Islam et al., 2024; Lukas and Umaternate, 2023). The language used to describe sex work remains a contested terrain within feminist and sociological debates. Terms such as “prostitute” and “prostitution” have been criticized for reproducing stigma and framing individuals as moral transgressors rather than workers embedded in structural inequalities (Smith and Mac, 2018). This study therefore deliberately adopts the term “sex work” to shift the analytical focus from individual deviance to labor, survival, and structural constraint, aligning with contemporary critical perspectives that emphasize the rights and lived experiences of sex workers. The internet and various digital applications now enable sex work activities to be carried out without face-to-face contact, creating a wider transaction space that transcends geographical boundaries, both regionally and globally (Awaludin, 2019). In this development, a number of instant messaging applications have become widely used for these activities, one of which is MiChat. This application is often used as a medium by covert sex workers because of its features, such as “people nearby,” which allows direct interaction between users within a certain radius, without the need for a formal meeting process (Juditha, 2022).

The use of the MiChat application as a medium for online sex work has come under scrutiny because of its nature, which allows for anonymity and high mobility (Buloto, 2024). These characteristics make it difficult for authorities to monitor and intervene. A number of studies show that this application has become a platform through which sexual services are negotiated and exchanged, reflecting the adaptive use of digital technologies within informal economies directly between users and service providers, without going through a platform that officially provides such services (Sukardi et al., 2021). This phenomenon reveals the dark side of digital technology, where ease of access and flexibility of communication can be used for activities that are socially contested, while also posing challenges for regulation, governance, and public protection, while also posing new challenges in terms of social supervision, law enforcement, and public protection.

Furthermore, the rise of online sex work is closely related to social welfare issues, both from an economic, psychological, and social perspective. From an economic standpoint, many individuals engage in online sex work as a response to structural constraints, including economic precarity, limited access to formal employment, and unequal livelihood opportunities and limited job opportunities, especially those living in structural poverty (Abidjulu, 2019). Meanwhile, psychologically, individuals engaged in online sex work, often positioned as victim-survivors, experience psychological pressure, stigma, and exposure to violence, the burden of social stigma, and trauma due to discrimination and violence experienced in their practice, which affects their quality of life and mental health (Tawakal and Imelda, 2022). On the other hand, socially, the existence of this phenomenon interacts with existing social norms and public perceptions of gender, sexuality, and morality, reflecting ongoing tensions between cultural expectations and socio-economic realities, interpersonal relationships, and public perceptions of the body and sexuality (Maulidani and Suminar, 2024).

The relevance of studies on online sex work becomes even more important when examined in a local context, such as in the city of Jayapura. As a major city in Eastern Indonesia that is undergoing progressive digital technology development, Jayapura faces its own challenges related to the spread of online sex work. Unfortunately, so far, in-depth research on this phenomenon in Jayapura is still very limited. Most existing studies focus more on major cities such as Jakarta or Surabaya, creating a research gap that needs to be filled immediately by comprehensive local studies (Mudjiyanto et al., 2024; Adli et al., 2023; Juditha, 2022).

The selection of Jayapura as the location for this study has a number of strategic reasons and has experienced rapid development, similar to several other major cities in Papua, such as Manokwari and Sorong (Rahawarin, 2025; Hamid et al., 2024). First, this city is currently in a phase of accelerated technological development, but on the other hand, it is still overshadowed by complex social and economic problems. Second, the use of applications such as MiChat in Jayapura shows trends and patterns that are not much different from other major cities, but with local characteristics that provide a new perspective. Therefore, this study aims to explore in depth the practice of online sex work in Jayapura and examine the social welfare aspects of its sex workers in order to understand how individuals navigate, negotiate, and sustain their participation in online sex work within conditions of structural constraint and limited social protection. The Ministry of Health (Kemenkes) estimates that there are approximately 1,700 female sex workers (PSP) in Jayapura City. Of this number, the Indonesian Family Planning Association (PKBI) Papua notes that 1,218 are hidden PSP. Then, there are approximately 495 PSP who have a Jayapura City KIT. Based on the above description, the researcher is interested in writing about a Social Welfare Review of the practice of online sex work through the MiChat application in Jayapura City.

This study addresses a critical gap in the literature on online sex work by examining how digitally mediated sexual labor is socially constructed and sustained within a social welfare context in Jayapura, Eastern Indonesia. Existing studies on online sex work largely emphasize legal regulation, technological facilitation, or moral discourse (Nicolas and Maharani, 2024; Juditha, 2022; Awaludin and Triana, 2019), while paying limited attention to how how individuals engaged in online sex work actively construct legitimacy, normalize participation, and negotiate their livelihoods, normalize participation, and negotiate welfare across economic, social, psychological, and spiritual dimensions. The core research problem concerns how online sex work through the MiChat application becomes a socially meaningful and as a socially negotiated livelihood strategy within contexts of stigma, regulatory ambiguity, and limited economic alternatives, and what this reveals about fragmented welfare realities in peripheral urban contexts. Conceptually, this study contributes by advancing the application of Berger and Luckmann's social construction theory to illuminate processes of micro-level legitimacy, digital-mediated normalization, and identity fragmentation that sustain online sex work. Empirically and analytically, the study reframes online sex work not merely as a stigmatized or moral issue, but as a manifestation of structural welfare gaps, thereby extending social welfare debates beyond income deprivation to include symbolic recognition, psychological security, and social protection in digital spaces. Rather than positioning individuals as sources of social problems, this study approaches them as subjects navigating constrained choices within unequal social, economic, and institutional structures. In this sense, individuals involved in online sex work are better understood as victim-survivors who actively negotiate survival, dignity, and social identity within structurally limited conditions.

## 2 Research method

The approach used in this study is a qualitative approach. Qualitative data collection was conducted through in-depth interviews with key informants and supporting informants using the interview method. This type of research is descriptive, which is research that provides a description in the form of an an overview of digitally mediated sex work practices through the MiChat application, focusing on how individuals navigate and experience these practices within their social welfare conditions, the welfare conditions of online sex workers, and the factors that drive and inhibit the social welfare of online sex workers in Jayapura City. The purpose of this type of research is to describe complex social realities in such a way that social welfare relevance can be achieved (Creswell and Poth, 2016).

The selection of informants in this study was conducted through purposive sampling with criteria that were consciously formulated and aligned with the analytical objectives of the study, namely to understand in depth the process of social construction of online sex work practices through the MiChat application from the perspective of the sex workers. Informants were selected based on several main considerations, including: (a) active engagement in online sex work through the MiChat application, understood as part of their livelihood strategies within constrained socio-economic conditions through the MiChat application, (b) duration of involvement that allows for reflection on relatively stable and repeated experiences, (c) diversity of socio-economic backgrounds to capture variations in meaning, motivation, and adaptation strategies, and (d) willingness of informants to engage in in-depth and open interviews. Thus, purposive sampling in this study was not intended to represent the population statistically, but rather to obtain depth of data and a variety of experiences that are conceptually relevant in analyzing the social construction, micro-legitimacy, and welfare dynamics experienced by individuals engaged in online sex work as they negotiate economic needs, social stigma, and everyday survival.

This study involved three purposively selected informants with distinct socioeconomic backgrounds to capture variations in motivation, meaning-making, and welfare consequences of engagement in online sex work through the MiChat application. As mentioned in Table 1, Informant A, a 22-year-old undergraduate student from a relatively secure upper-class background, had been involved for approximately 1 year, motivated primarily by curiosity and peer influence, and perceived the activity as a temporary and exploratory strategy to support her lifestyle needs within a flexible and accessible economic opportunity rather than to meet basic needs, while maintaining secrecy from family and receiving limited legitimacy from close friends alongside regular reproductive health check-ups at a private clinic. In contrast, Informant B, a 23-year-old freelance café worker representing the middle class, had engaged in online sex work for about 2 months as an a necessary economic strategy to cope with financial pressure and fulfill caregiving responsibilities to cope with financial pressure and fulfill responsibilities toward her child, conducting the practice in strict secrecy from her family, relying on tolerance from boarding house peers, and routinely accessing reproductive health services as a preventive measure. Meanwhile, Informant C, a 45-year-old housewife from a lower socioeconomic background, had been involved for approximately 5 months, driven by urgent household subsistence needs within conditions of limited access to stable employment and economic resources, and uniquely received domestic legitimacy from her spouse while concealing the activity from extended family to avoid stigma, carrying out transactions in relatively safe private spaces and regularly visiting a community health center. Taken together, these profiles illustrate that online sex work via MiChat is not a homogeneous practice but is shaped by class position, family roles, and sources of legitimacy, resulting in differentiated meanings, coping strategies, and welfare vulnerabilities among informants.

**Table 1:** Informant profiles.

Informant	Age	Gender	Socioeconomic status and occupation	Duration of involvement	Primary motivation
Informant A	22	Female	Upper class; undergraduate student	±1 year	Curiosity and peer influence
Informant B	23	Female	Middle class; freelance café worker	±6 months	Economic pressure and responsibility toward child
Informant C	45	Female	Lower class; housewife	±8 months	Fulfillment of basic household needs

The limited number of informants in this study is a consequence of methodological choices that emphasize depth of qualitative analysis over breadth of empirical representation. This research departs from an interpretive paradigm that focuses on the exploration of subjective meanings, lived experiences, and social processes constructed through interaction. Therefore, the sample size is determined by achieving data saturation (theoretical saturation), rather than by quantitative considerations. Data are considered to have reached saturation when follow-up interviews no longer yield new categories of meaning but instead repeat previously emerging narrative patterns related to structural economic pressures, social legitimacy, psychological experiences, and constrained future opportunities, and future aspirations. Therefore, three informants are considered analytically adequate because each represents a different socioeconomic configuration but demonstrates a relatively consistent pattern of social construction, allowing the researcher to conduct an in-depth and focused thematic analysis.

To enhance methodological transparency, this study further clarified the data collection design, specifically the use of semi-structured interviews guided by a thematic interview framework. The interview guide was developed to explore key dimensions relevant to the research objectives, including participants' pathways into online sex work, including the structural, economic, and social conditions shaping their entry, strategies used by participants to negotiate safety, income, and control within their interactions with clients, perceived risks shaped by structural vulnerabilities, including stigma, health exposure, and safety concerns, forms of social legitimacy within their immediate environment, and the subjective meanings attached to their involvement. This approach ensured flexibility in capturing personal narratives and consistency in addressing core analytical themes, thereby enhancing the credibility and traceability of the data collection process.

Furthermore, although the number of informants is limited, this study justifies its sampling strategy within an interpretive qualitative framework that prioritizes depth over statistical representativeness. Informants were purposively selected to reflect variation in socioeconomic backgrounds, enabling comparative insights across different lived experiences. The adequacy of the sample is determined by thematic saturation, indicated by recurring patterns in narratives related to motivation, legitimacy, and welfare experiences. Thus, the small sample size is not treated as a limitation alone, but as a deliberate methodological strategy to generate in-depth and contextually grounded understanding of online sex work practices in Jayapura.

Data collection was conducted through semi-structured interviews, allowing flexibility in exploring informants' personal narratives while maintaining a focus on key themes relevant to the research objectives. Interview questions were developed gradually, following the informants' experiences and reflections, ensuring a dialogic data collection process that was responsive to the field context. Data analysis was conducted inductively through initial coding, theme grouping, and analytical category development, which were then linked to Berger and Luckmann's social construction theory framework. The analysis process was not merely mechanical, following a reduction-presentation-conclusion procedure, but also reflective, considering how meaning, legitimacy, and identity are constructed, negotiated, and maintained by participants as they navigate online sex work within their everyday social and economic realities. With this approach, empirical data serves as the primary basis for constructing theoretical interpretations, rather than simply as an illustration of the theory.

The analysis technique uses three stages, including data reduction, data display, and conclusion drawing (Miles and Huberman, 1992). In the data reduction stage, transcripts are immediately made from the interviews. Next, the observations are coded to create categories and themes based on the issues and research questions. Then, in the data submission stage, the data is grouped according to themes and/or main issues, namely the form of online sex work using the MiChat application. In the final stage, the research results are described in a report and concluded using more basic, objective techniques, referring to efforts to understand the data more extensively and deeply.

Given the sensitivity of the research topic and the structurally vulnerable position of the informants, particularly in relation to stigma, gender inequality, and economic precarity, ethical aspects were a primary concern throughout the research process. All informants were given a clear explanation of the research objectives, interview procedures, and potential risks, and participation was based on informed consent. Informants' identities were fully protected through the use of pseudonyms and the omission of information that could lead to personal identification. Interviews were conducted with due regard for the informants' psychological comfort, including allowing them to decline to answer certain questions or terminate the interview at any time. The researchers also attempted to minimize the risk of stigma and emotional distress by maintaining data confidentiality and avoiding judgmental approaches. Thus, this research not only strives to meet academic standards but also upholds the principle of protecting research subjects in the context of sensitive social issues.

This study recognizes that the researchers' academic background and social welfare perspective may influence data interpretation, particularly given the sensitive nature of online sex work. To minimize bias, reflexivity was applied throughout the research process by critically reflecting on assumptions, prioritizing participants' own meanings, and maintaining a non-judgmental stance during interviews. Open-ended questions were used to allow informants to articulate their experiences and welfare priorities in their own terms. Analytical interpretations were continuously cross-checked against empirical narratives to ensure that findings remained grounded in participants' accounts rather than external moral frameworks, thereby strengthening the credibility and ethical integrity of the study. The study explicitly avoids framing participants as deviant actors, instead positioning them as individuals navigating constrained choices within unequal structural conditions. In this research, participants are approached not as objects of social problems, but as active agents and, in many cases, victim-survivors who negotiate survival, dignity, and wellbeing within structurally limited opportunities.

## 3 Results

### 3.1 Forms of online sex work through MiChat

The three informants showed relatively similar patterns in conducting online sex work through the MiChat application. The interaction process generally began with the use of the chat feature as a space for negotiation between the informant and the customer. During these conversations, various aspects of the transaction are discussed, ranging from the type of service offered and the price to the payment mechanism that will be used. This pattern of interaction is evident in the following account:

“*Usually, I just wait for messages through MiChat. We talk about the price first—normally around IDR 300,000 for standard service, and it can go up to IDR 700,000 or even IDR 800,000 depending on what they ask, then discuss what kind of service they want, and then decide where to meet. It feels fast and simple, like ordering something online.”* (Informant A)

This account confirms the structured and routinized nature of transactions mediated through the platform, where digital interaction simplifies negotiation while maintaining a degree of anonymity. Similarly, another informant emphasized the importance of clarity and risk management:

“*I prefer to discuss everything clearly in chat before meeting, especially the price and time. I don't want any misunderstanding because it can be risky.”* (Informant B)

This reflects how digital mediation is not merely a tool for communication, but also a mechanism for minimizing uncertainty and perceived risk. Furthermore, spatial control emerges as an important element in transaction dynamics:

“*I always choose the place myself because I feel safer that way. If the customer insists on another place, I will think twice before accepting.”* (Informant C)

The three informants showed relatively similar patterns in conducting online sex work through the MiChat application. The interaction process generally began with the use of the chat feature as a space for negotiation between the informant and the customer. During these conversations, various aspects of the transaction are discussed, ranging from the type of service offered and the price to the payment mechanism that will be used. Based on the information obtained, the price for standard services is generally set at IDR 300,000, while the price range for full services is between IDR 700,000 and IDR 800,000. The payment mechanism varies, either in cash (cash on delivery or COD) or through bank transfer. However, the majority of payments are made during face-to-face meetings, which indicates a preference for a payment system that is considered safer and reduces the risk of fraud. Online sex work mediated through MiChat reflects how individuals adapt digital technologies to navigate economic opportunities within informal and constrained labor conditions in utilizing chat features for quick and low-risk negotiations. Digital technology reconstructs this practice into a form of digitally mediated labor that is less visible and more difficult to regulate within existing institutional frameworks, both socially and legally (Perawati and Dompak, 2024; Natalis and Djohan, 2025).

The determination of the meeting location is generally under the control of the informants, although the nature of this control can be said to be limited because it still considers customer demand and security factors. Interestingly, the pattern of demand intensity shows a distinctive temporal dynamic. Demand for services tends to increase at the beginning of the month and on important days, such as religious celebrations or long holidays. This phenomenon can be interpreted as a seasonal pattern in online sex work practices, which is closely related to the community's economic cycle and an increase in customer consumption power during certain periods. Thus, online sex work practices not only reflect individual interactions but are also influenced by broader structural factors, such as monthly economic dynamics and social celebrations. This phenomenon shows that the increased demand for online sexual services at the beginning of the month and during holidays reflects seasonal patterns related to consumption behavior (Markey and Markey, 2013). In addition, economic conditions such as recession also affect the supply of sex workers (Goehring, 2025).

### 3.2 Social construction of sex work online through MiChat

#### 3.2.1 Externalization

The externalization stage refers to the process by which individuals express subjective conditions and structural pressures into concrete social actions. In this study, informants' involvement in online sex work through the MiChat application was an active response to the life circumstances they faced. The externalization stage refers to how individuals translate subjective conditions into concrete actions. The underlying motivation for involvement is clearly articulated by the informant:

“*At first, I was just curious. My friends talked about it, and I thought maybe I could try it just for fun and extra money.”* (Informant A)

This statement highlights how individual action emerges not from structural pressure alone, but also from peer influence and lifestyle orientation. In contrast, economic necessity becomes a dominant driver for another informant:

“*I didn't really want this, but I needed money for my child. My job at the café was not enough, so I had no other option.”* (Informant B)

This demonstrates that participation is often framed as a constrained choice shaped by economic responsibility. A more structural urgency is reflected in the following account:

“*I joined because my family needed money. It was not my choice, but I had to survive and help my household.”* (Informant C)

This reinforces the interpretation that online sex work functions as an adaptive response to structural vulnerability rather than purely individual decision-making. Informant A externalized curiosity and lifestyle urges by using MiChat as a space for social and economic exploration. She viewed the application as “easy, fast, and non-binding,” allowing her to earn additional income without being tied to formal employment. At this stage, engagement in online sex work was not yet internalized as a stable identity, but rather as a social experiment emerging from interactions with her social circle. In contrast to Informant A, Informant B externalized economic pressures and family responsibilities into her decision to use MiChat. The need to regularly send money to her child was the dominant factor driving her involvement. In her narrative, participation in online sex work was understood as a rational and necessary strategy to cope with economic constraints to overcome limited income from informal work in the cafe sector. Externalization in this context was not exploratory, but rather instrumental and oriented toward fulfilling social obligations as a mother.

Meanwhile, Informant C externalized the conditions of household poverty and limited economic access as a survival strategy. Her involvement in online sex work through MiChat arose from a pressing need to meet basic family needs, with direct support from her husband. At this stage, participation is shaped less by autonomous choice and more by structural constraints and limited economic alternatives, but as an expression of structural urgency. Thus, the externalization stage demonstrates that online sex work is a direct expression of the social, economic, and relational conditions experienced by each informant.

The externalization stage in this study demonstrates how the subjective conditions and structural pressures of the informants are expressed in concrete actions, such as involvement in online sex work through the MiChat application. Informant A externalizes curiosity, the influence of social circles, and a consumerist lifestyle orientation into using MiChat as a space for economic exploration that is considered flexible, anonymous, and non-binding, so that online sex work is understood as an experimental activity that has not yet been attached to personal identity. In contrast, Informant B externalizes economic pressures and family responsibilities—particularly the obligation to send money to children—as a rational decision to use MiChat as an alternative source of income that is faster than the informal work she does. Meanwhile, Informant C externalizes the urgency of the household economy and limited access to work as a survival strategy, with involvement in online sex work even gaining domestic legitimacy from her husband. This difference shows that at the externalization stage, online sex work does not emerge as an established reality, but rather as an initial expression of different needs, pressures, and life orientations, where engagement in online sex work functions as an adaptive response to socio-economic conditions to the social and economic conditions experienced by each informant.

#### 3.2.2 Objectivation

The objectivation stage occurs when actions that were initially individual and situational begin to form repetitive, stable patterns, and are perceived as objective reality. In this study, online sex work via MiChat underwent a process of objectification through transaction routines, standardized rates, and meeting mechanisms that were relatively uniform among informants. All three informants used the MiChat chat feature as a negotiation space, with standardized service prices (IDR 300,000 for standard service and IDR 700,000–800,000 for full service). This pattern indicates that online sex work practices have undergone informal institutionalization within everyday social and economic interactions, accepted as "normal business practices. Objectification is also reinforced by the emergence of micro-legitimizations within the informants' environment. The process of normalization is reflected in the informant's narrative:

“*It became normal for me after some time. I already know how to set the price and how to deal with customers.”* (Informant A)

This indicates that repeated interaction transforms initially situational practices into routinized patterns perceived as normal. Micro-level legitimacy is also evident in social surroundings:

“*My friends at the boarding house know about it, and they don't judge me. That makes me feel more comfortable to continue.”* (Informant B)

This suggests that normalization is sustained through localized tolerance rather than broader societal acceptance. In some cases, legitimacy is reinforced within intimate relationships:

“*My husband knows and supports me. He understands our situation, so I don't feel alone doing this.”* (Informant C)

This highlights how informal social institutions contribute to the objectification of the practice as a socially negotiable reality. Informant A received normalization from peers who were aware of and did not object to the activity. Informant B received tolerance from her roommates, while Informant C received direct support from her husband. This support serves as a social mechanism that reduces moral resistance and reinforces the perception that the practice is negotiated and tolerated within specific social environments. Thus, online sex work is no longer perceived solely as stigmatized, but as an economic activity that is “tolerated” under certain conditions.

Furthermore, access to reproductive health services plays a role in the objectification process. All three informants regularly undergo health check-ups at clinics or community health centers, which indirectly contributes to the continuation of online sex work. These health services become part of an institutional structure that, while not normatively supportive, enables individuals to sustain their participation under more manageable health-related conditions with perceived more manageable risks. At this stage, online sex work has evolved from situational engagement into a routinized and socially embedded practice into an institutionalized social reality perceived as objective by the actors.

The objectivation stage is evident when the initially situational practice of online sex work begins to experience repetition, stabilization, and informal institutionalization, thus being perceived as an objective reality. The three informants demonstrated a relatively uniform pattern in carrying out the practice, ranging from the use of the MiChat chat feature as a negotiation space, setting rates that tend to be standardized, payment mechanisms (cash or transfer), to determining meeting locations that consider security aspects. This recurring pattern indicates that online sex work has transformed into a “regular” and predictable practice, no longer simply a spontaneous individual act. The objectivation process is further reinforced by the micro-legitimacy that informants receive from their immediate environment, such as peer support for Informant A, tolerance from roommates for Informant B, and direct support from partners for Informant C, which collectively serve to reduce perceived social tension and enable continued participation within specific social contexts and frame the practice as something that is acceptable in certain contexts. Furthermore, routine access to reproductive health services also acts as an institutional structure that enables the continuation of online sex work, as it provides a sense of security relative to health risks. Thus, through routine, limited social support, and connections to health institutions, online sex work is objectified as an informally institutionalized social reality perceived as “real” by the actors.

#### 3.2.3 Internalization

The internalization stage refers to the process by which objectified reality is reabsorbed into an individual's consciousness and forms subjective meaning and self-identity. In this study, all three informants demonstrated an internalization process characterized by an ambivalence between pragmatic acceptance and moral rejection. The internal conflict experienced by the informant is expressed as follows:

“*I don't see this as who I am. It's just something temporary. I still have my life as a student*.” (Informant A)

This reflects a distancing strategy where identity is separated from economic activity. However, for other informants, internalization is marked by stronger moral tension:

“*Sometimes I feel guilty, especially when I pray. But I tell myself I'm doing this for my child.”* (Informant B)

This illustrates the coexistence of moral conflict and pragmatic justification. A deeper ambivalence is evident in the following account:

“*I feel torn inside. I know this is wrong, but I have no other way right now*.” (Informant C)

This demonstrates that internalization is not a process of full acceptance, but an ongoing negotiation between structural necessity and moral values. Informant A internalized online sex work as a temporary activity that “wouldn't necessarily continue,” thus maintaining a distance between her economic activities and her personal identity as a student. This strategy enabled her to maintain a positive self-image while navigating tensions between personal aspirations and prevailing social expectations. Informants B and C demonstrated a more complex internalization, characterized by recurring psychological and moral conflicts. Both recognized that online sex work conflicted with religious values and social norms, yet they nonetheless interpreted it as a sacrifice for their families. Guilt, anxiety, and fear of stigma became part of their daily experiences as a consequence of this life circumstances shaped by economic necessity and social constraints. Continuing religious practices served as an internal mechanism to mitigate moral dissonance and maintain psychological balance.

Internalization was also evident in how informants viewed the future. All three expressed aspirations to leave online sex work and build new identities through more socially acceptable employment or businesses. However, limited capital and access to economic empowerment programs make these aspirations difficult to achieve. This demonstrates that internalization is not final, but rather a continuous negotiation within the tension between structural realities and subjective expectations. Thus, engagement in online sex work is not experienced as a permanent identity, but as a phase of life interpreted ambiguously and temporarily.

The internalization stage is reflected in how informants reabsorb the objectified reality of online sex work into their subjective consciousness, forming ambivalent meanings, attitudes, and self-identities. Informant A internalized online sex work as a temporary activity separate from her primary identity as a student, thus maintaining a symbolic distance between her economic actions and the self-concept she sought to maintain. Informants B and C demonstrated a more conflict-ridden internalization, where online sex work was interpreted as a sacrifice for the sake of family as well as a source of guilt, anxiety, and fear of social stigma. Moral and religious conflicts became part of everyday internal experiences, managed through spiritual strategies such as continuing to practice religious services as a mechanism to reduce value dissonance and maintain psychological balance. Internalization was also evident in the informants' future orientations, who consistently expressed a desire to leave online sex work and build a new identity through more socially acceptable work or business, although these aspirations were hampered by limited capital and access to economic empowerment programs. Thus, internalization shows that online sex work is not fully accepted as a permanent identity, but rather interpreted ambiguously as a temporary phase of life that is continuously negotiated amidst the tension between structural reality and subjective expectations.

### 3.3 Social welfare conditions of the actors

#### 3.3.1 Economic dimension

Differences in the orientation of income use from online sex work practices were evident among the three informants. The economic orientation of the informant is illustrated in the following statement:

“*I use the money mostly for my personal needs, like shopping or hanging out. It's more about lifestyle*.” (Informant A)

This demonstrates how income utilization is shaped by lifestyle aspirations and symbolic consumption rather than basic necessity, reflecting a relatively secure socioeconomic position. In contrast, economic responsibility becomes central in the following account:

“*Most of the money I earn goes to my child. That is my priority.”* (Informant B)

This indicates that participation is embedded within caregiving obligations and reflects a survival-oriented economic rationality linked to family responsibility. A more urgent subsistence orientation is evident below:

“*This money is for daily needs—food, electricity, everything. Without it, we cannot survive.”* (Informant C)

This highlights that online sex work functions as a critical livelihood strategy under conditions of economic precarity. Taken together, these variations demonstrate that economic motivations are not homogeneous, but are shaped by class position, social roles, and differing levels of structural vulnerability. Informant A allocated his income primarily to meet consumptive lifestyle needs, such as entertainment, fashion, or non-essential social activities. In contrast, Informant B uses her income more functionally, namely to fulfill financial obligations to her child who is being cared for by her parents, thus reflecting a sense of family responsibility. Meanwhile, Informant C uses her income to support basic daily household needs, reflecting a more urgent and survival-oriented economic situation.

The variation in income utilization patterns indicates a differentiation in economic orientation influenced by each individual's social background and living conditions. On the one hand, online sex work serves as a means through which individuals navigate different economic needs of fulfilling tertiary needs and lifestyle (Informant A), while on the other hand, it becomes a vital economic instrument for family survival and basic needs (Informants B and C). These findings confirm that financial motivations and orientations are not homogeneous, but are greatly influenced by age, social role, and economic pressures. Thus, analysis of income utilization can reflect the complexity of the socio-economic structure that underlies individuals' engagement in online sex work within their socio-economic context. This shows that the economic orientation of online sex workers reflects adaptive strategies to structural pressures and the social roles they play (Bernstein, 2007). The use of income also shows attachment to affective relationships and family responsibilities (Zelizer, 2005).

#### 3.3.2 Social dimension

The strategies for managing secrecy appear to differ among the informants. Informants A and B consistently hide their involvement in online sex work from their extended families, with the aim of avoiding stigma, conflict, and potential social rejection. The strategy of managing social stigma is evident in the informant's account:

“*My family doesn't know, and I don't want them to know. They would be very disappointed.”* (Informant A)

This indicates the presence of strong normative pressures that require individuals to conceal their activities to maintain social identity. Similarly, another informant described selective disclosure:

“*I hide this from my family. Only some close friends know, and they understand my situation.”* (Informant B)

This reflects the existence of limited social acceptance within close networks, which partially mitigates stigma while maintaining broader secrecy. A different pattern emerges in the following account:

“*My husband supports me, but we keep it secret from others because people will judge us.”* (Informant C)

This highlights how legitimacy can exist at the micro-level within intimate relationships, while stigma persists at the societal level. Overall, these findings indicate that online sex work is sustained through fragmented social support, where acceptance is localized and conditional rather than institutionalized. This attitude illustrates the symbolic alienation experienced by individuals when their activities do not conform to prevailing social norms. In contrast, Informant C received support from her husband, even though her involvement was kept secret from her extended family. This internal support served as a form of domestic legitimacy that provided a safer psychological space, while reducing the moral pressure that usually accompanies sex work.

However, in general, the social support received by the informants remains limited. The lack of a supportive social network makes them vulnerable to feelings of social and psychological isolation. This isolation can lead to emotional burdens, feelings of alienation, and mental instability as a result of carrying out activities that are socially contested within prevailing cultural norms. In the context of Informant C, the support of her husband does alleviate some of the social and moral burden she bears, but it does not completely eliminate external stigma. This confirms that in the context of online sex work participation, the dynamics of social support play a crucial role in influencing the extent to which individuals are able to manage the psychosocial pressures inherent in such activities. This shows that the strategy of hiding one's identity reflects the strong pressure of social norms against stigmatized behavior (Goffman, 1963). The lack of social support increases the risk of psychological disorders due to isolation (Thoits, 2011).

#### 3.3.3 Psychological dimensions

Psychological experiences among informants reveal a complex interplay between emotional vulnerability and situational uncertainty in their daily interactions. These experiences are not only shaped by immediate transactional risks but also by deeper moral tensions and exposure to potential harm. The psychological pressure experienced by the informant is captured in the following statement:

“*Sometimes I feel anxious before meeting someone new because I don't know what will happen.”* (Informant A)

This illustrates uncertainty as a constant emotional burden in transactional encounters. Another informant emphasized fear and vulnerability:

“*I feel scared and worried, especially about violence or being treated badly.”* (Informant B)

This highlights the inherent risks embedded in the interaction. More severe impacts are reflected in the following account:

“*There were times when I felt very stressed and even traumatized because of how customers treated me.”* (Informant C)

This indicates that online sex work is not only an economic activity, but also a site of psychological vulnerability and potential trauma. All three informants indicated significant psychological pressure as a consequence of their involvement in online sex work. This pressure generally manifested itself in the form of guilt, anxiety, and fear that arose repeatedly. Guilt was mainly related to the conflict between their activities and their moral and religious beliefs. Anxiety arises from uncertainty about the safety of transactions, health risks, and the potential for their identities to be revealed to their families or the wider community. Meanwhile, fear is often related to the possibility of encountering aggressive customers, acts of violence, or situations beyond their control.

In addition to general emotional pressure, some informants also experienced more specific traumatic experiences. These traumas include physical violence, verbal abuse, and violations of agreements regarding condom use. Such situations not only increase vulnerability to short-term psychological disorders but also have the potential to cause long-term effects, such as symptoms of post-traumatic stress disorder (PTSD), depression, and decreased self-confidence. This condition shows that online sex work should not be understood solely as an economic activity, but also a space for interaction that is fraught with risks to the mental wellbeing of individuals engaged in this form of work. Thus, the psychological dimension is a crucial aspect in understanding the complexity of the informants' experiences, which cannot be separated from the structural and social vulnerabilities they face.

This confirms that psychological pressure in online sex work is greatly influenced by moral value conflicts and structural insecurity (Lutnick, 2016). In addition, the traumatic experiences of the informants reflect the findings of Farley et al. (1998) that women in the online sex industry have a high prevalence of PTSD and depression symptoms.

#### 3.3.4 Spiritual dimension

The spiritual dimension reflects an ongoing negotiation between deeply held religious values and the practical realities of economic survival. Rather than representing a clear moral alignment, informants' experiences indicate a persistent internal dialogue shaped by guilt, faith, and the need for emotional stability. The moral and spiritual conflict is reflected in the informant's expression:

“*I still pray, but sometimes I feel like I'm not a good person because of what I do.”* (Informant A)

This highlights the coexistence of religious practice and moral dissonance, indicating that spiritual identity remains important despite behavioral contradiction. Another informant expressed a similar internal struggle:

“*I always ask God for forgiveness. I know this is not right, but I have no choice.”* (Informant B)

This suggests that religiosity functions as a mechanism to negotiate guilt and justify actions under constrained circumstances. A deeper emotional reliance on spirituality is evident in the following account:

“*Prayer helps me feel calmer. It's the only way I can deal with my guilt.”* (Informant C)

This demonstrates that religious practices serve not only as moral reference points but also as coping strategies for psychological distress. Although the three informants realized that their engagement in online sex work was experienced as being in tension with religious teachings, they still tried to worship and pray regularly. This phenomenon reveals a strong internal contradiction, namely an inner conflict between the demands of life on the one hand and moral and religious values on the other. The religious practices that continue to be carried out serve as a psychological compensation mechanism, in which prayer and worship rituals are perceived as a way to alleviate guilt while maintaining a spiritual relationship with God.

This kind of inner conflict can be understood as a form of cognitive dissonance, when individuals hold on to moral values that they believe to be right but at the same time do things that go against them. In this context, worship and prayer not only represent an expression of faith, but also become a strategy to maintain emotional balance and provide moral legitimacy that allows informants to survive in an ambivalent situation. Thus, religiosity plays a dual role: on the one hand, it is a source of psychological tension, but on the other hand, it also provides a space for catharsis and spiritual resilience amid the economic hardship and social pressures they face.

This contradictory phenomenon shows that religious practices can function as a coping mechanism in morally stressful conditions. This is in line with Pargament's (2001) findings, which emphasize that religiosity is often used to manage psychological stress in conflict-ridden situations. In addition, a study by Sari et al. (2025) reinforces that individuals involved in socially stigmatized forms of work maintain worship as a form of defense against anxiety and guilt.

#### 3.3.5 Health dimension

Health-related practices among informants demonstrate a level of awareness regarding the risks associated with online sex work, particularly in relation to reproductive health. However, this awareness is mediated by structural constraints, including limited access to affordable and inclusive healthcare services. Awareness of health risks is articulated by the informant as follows:

“*I regularly go to a clinic to check my health because I know the risks*.” (Informant A)

This indicates a preventive orientation among informants, reflecting an awareness of vulnerability in transactional sexual encounters. Another informant emphasized the importance of health monitoring:

“*Health is important, so I always try to do check-ups even though it costs money.”* (Informant B)

This highlights the economic burden associated with maintaining health, particularly in the absence of accessible and inclusive services. Structural limitations are further reflected below:

“*I go to the community health center to make sure I am safe, but sometimes it's difficult because of the cost.”* (Informant C)

All three informants demonstrated a high level of awareness of the importance of reproductive health. This is reflected in their habit of regularly undergoing health checkups at various health facilities, ranging from specialized clinics and reproductive health centers to community health centers. This form of livelihood of routine check-ups shows preventive efforts to minimize the risk of sexually transmitted diseases (STDs), while also reflecting an awareness of the structural vulnerabilities associated with online sex work.

However, this awareness does not fully guarantee optimal protection. Limited access, both in terms of the availability of medical services that are friendly to vulnerable groups and the costs that must be borne, remains a major obstacle. In addition, the varying quality of services, which are often not integrated with psychosocial support programs, results in incomplete health protection. This situation is exacerbated by the possibility of violations of condom use agreements, which further increases the risk of STI transmission and other infectious diseases.

Thus, despite the awareness and initiative of the informants to maintain their reproductive health, structural factors such as limited services and low public health system support continue to make them a highly vulnerable group. This situation underscores the importance of more inclusive health policy interventions, whether through improving the quality of reproductive services, expanding access, or strengthening health education for individuals involved in online sex work. Individual awareness of the importance of maintaining reproductive health has not been matched by adequate structural support. Limited friendly services and minimal integration of psychosocial support mean that protection remains weak (Habib et al., 2024). In addition, stigma and a lack of digital outreach strategies narrow access to preventive services (Yunus and Mahendra, 2020).

### 3.4 Driving and hindering factors

The driving factors behind informants' involvement in online sex work are essentially rooted in urgent financial needs, both to support daily life and to fulfill family obligations. The duality of motivation and constraint is expressed in the informant's narrative:

“*It started as lifestyle, but sometimes I also feel afraid of what people will think if they find out.”* (Informant A)

This reflects the coexistence of voluntary engagement and social anxiety, indicating that even non-economic motivations are accompanied by stigma-related concerns. Economic necessity remains a dominant driving factor, as reflected below:

“*I do this because I need money, but at the same time I feel guilty and worried.”* (Informant B)

This illustrates the tension between economic survival and moral conflict, which shapes continued participation. A more structural burden is evident in the following account:

“*This helps my family survive, but it also gives me a heavy emotional burden.”* (Informant C)

Specifically, Informant A showed a different pattern, where the main motivation was more related to a consumptive lifestyle and the urge to maintain social status within their social circle. This difference shows that economic motivation is not homogeneous, but is influenced by the social conditions, age, and life orientation of each individual.

On the other hand, there are a number of inhibiting factors that cause moral and psychological dilemmas. Moral-spiritual burdens occupy an important position, because sex work is considered contrary to religious norms and social values. In addition, health risks, particularly those related to sexually transmitted diseases, were a consistent concern in the experiences of all three informants. Not only that, instability in relationships with customers—in terms of frequency, consistency of payment, and the potential for violence—often added to the uncertainty of this practice. The potential for social stigma attached to the identity of online sex workers further exacerbates the psychological burden, forcing informants to maintain secrecy in order to protect themselves from social rejection.

Thus, engagement in online sex work is the result of a tug-of-war between economic drivers and moral, health, and social barriers. This complexity makes the practice not just an economic activity, but also a space of ongoing negotiation shaped by economic needs, social expectations, and structural constraints involving values, identity, and multiple layers of risk. This phenomenon shows that engagement in online sex work is not solely driven by economic pressures, but also by socio-cultural dynamics that shape individual identity and symbolic needs (Sonjaya, 2025). Furthermore, the psychological burden arising from stigma and moral dilemmas is in line with the findings of Sanders et al. (2017), which emphasize the importance of a more humanistic approach in understanding the experiences of sex workers in the digital context.

### 3.5 Future hopes and aspirations

The findings of this study demonstrate that online sex work through the MiChat application in Jayapura constitutes a multidimensional social welfare issue shaped by local institutional gaps rather than a purely moral or legal problem. Although online sex work temporarily fulfills economic needs, informants consistently expressed aspirations to transition out of online sex work, which are constrained by limited access to employment opportunities, business capital, and targeted empowerment programs. This indicates that existing local welfare interventions implemented by the Dinas Sosial Kota Jayapura and the Dinas Pemberdayaan Perempuan dan Perlindungan Anak Provinsi Papua have not effectively reached vulnerable women engaged in digitally mediated informal economies. From a social construction perspective, this institutional absence enables online sex work to gain micro-legitimacy as a survival strategy in everyday life. Therefore, local policy responses should prioritize exit-oriented economic empowerment through vocational training, small-scale entrepreneurship assistance, and conditional livelihood support that are explicitly designed for women seeking to transition out of online sex work within the urban labor context of Jayapura.

Furthermore, the study reveals that health and social protection services operate in a fragmented manner at the local level. While informants routinely access reproductive health services through Puskesmas Kota Jayapura and clinics coordinated with the Dinas Kesehatan Kota Jayapura, these services remain weakly integrated with psychosocial counseling and trauma-informed care, despite widespread experiences of anxiety, fear, and exposure to violence. This fragmentation points to a governance gap in which health institutions function primarily as medical service providers rather than as entry points to comprehensive social protection. In addition, the persistence of online sex work is reinforced by micro-legitimacy within close social networks, suggesting the need for community-based interventions involving local civil society organizations and faith-based groups coordinated with the Badan Perencanaan Pembangunan Daerah Provinsi Papua. Effective policy responses in Jayapura therefore require multisectoral coordination among social services, health institutions, women's empowerment agencies, and planning bodies to address online sex work as a structurally produced welfare vulnerability, consistent with social construction theory's emphasis on institutional meaning, legitimacy, and access.

## 4 Discussion

This research demonstrates that online sex work mediated through the MiChat application in Jayapura should not be understood as a homogeneous or purely individual phenomenon, but rather as a socially constructed practice embedded within structural inequalities and institutional limitations. Drawing on Berger and Luckmann (1966), the processes of externalization, objectivation, and internalization identified in this study are shaped not only by subjective meanings but also by broader socio-economic conditions that constrain and enable individual action. Informant A's engagement reflects the normalization of digital-mediated intimacy within youth culture, while Informants B and C illustrate how participation is closely tied to economic precarity, caregiving responsibilities, and restricted access to formal employment. Thus, the practice emerges not as a matter of individual deviance, but as a situated response within unequal social structures. This study also responds to critical debates on terminology, recognizing that the use of stigmatizing labels can obscure structural inequalities and reinforce marginalization. Following Smith and Mac (2018), the analysis refrains from moralizing language and instead positions individuals as subjects navigating constrained choices within unequal social systems.

Socioeconomic differentiation plays a central role in shaping both the meaning and function of online sex work. The findings indicate that participation is stratified along class lines, where individuals with relatively secure economic backgrounds maintain symbolic distance from the practice, while those in more precarious conditions experience deeper entanglement with it. This supports Bernstein's (2007) argument that sexual labor is structured by class position, relational dynamics, and economic necessity. In this context, online sex work should be understood as a differentiated survival strategy rather than a uniform economic activity, reflecting the uneven distribution of opportunities and resources in society.

From a social perspective, the persistence of online sex work is sustained through fragmented forms of legitimacy that operate at the micro-level. Informants rely on selective acceptance within intimate networks while simultaneously managing stigma in broader social contexts. This aligns with Goffman's (1963) concept of stigma, where individuals actively negotiate identity by controlling information and managing social visibility. Importantly, this pattern does not indicate a failure of individual morality, but rather reflects exclusionary social norms and the absence of inclusive social protection mechanisms that could provide alternative pathways for economic and social participation.

Psychological distress emerges as a structurally conditioned outcome rather than an individual pathology. Feelings of anxiety, guilt, and fear reported by informants are closely linked to economic insecurity, exposure to violence, and persistent social stigma. As noted by Farley et al. (1998), women involved in the sex industry frequently experience high levels of trauma and psychological distress, while Lutnick (2016) emphasizes that such experiences are embedded within broader systems of vulnerability and marginalization. Therefore, psychological conditions should be understood as socially produced consequences of precarious labor conditions, rather than as inherent attributes of individuals involved in sex work.

The spiritual dimension further illustrates how individuals navigate complex moral landscapes under structural constraints. The coexistence of religious practice and participation in online sex work reflects an ongoing negotiation between normative values and material necessity. Drawing on Pargament (2001), religiosity can function as a coping mechanism that enables individuals to manage stress and maintain emotional stability in situations of moral conflict. In this sense, moral ambivalence does not signify deviance, but rather reveals how individuals actively reconcile competing value systems within constrained life circumstances.

Gender also emerges as a critical structural dimension that shapes vulnerability and participation in online sex work. The experiences of the informants indicate that women disproportionately bear the burden of economic responsibility, caregiving, and social stigma, reflecting broader patterns of gender inequality. This aligns with Sanders et al. (2017), who argue that digital sex work is deeply embedded within gendered power relations and labor inequalities. In the Indonesian context, these dynamics are further intensified by cultural norms surrounding femininity, morality, and sexuality, which place additional pressure on women while limiting their access to alternative economic opportunities. Consequently, online sex work should be understood as part of the feminization of economic vulnerability in informal and digital labor markets.

The institutional dimension reveals a significant governance gap that reinforces the persistence of online sex work. Although informants demonstrate awareness of health risks and actively access reproductive health services, these services remain fragmented and are not integrated with broader systems of social protection, economic empowerment, or psychosocial support. As argued by Marmot (2005), health outcomes are fundamentally shaped by social determinants, including access to resources and institutional support. In this case, the limited and disconnected nature of health services reflects broader structural inequalities that fail to address the multidimensional needs of individuals engaged in precarious labor.

Furthermore, the Indonesian regulatory context contributes to the structural conditions that shape online sex work. The criminalization and stigmatization of sex work, as highlighted by Awaludin (2019), push the practice into informal and digital spaces where regulation is limited and vulnerability is heightened. Digital platforms such as MiChat provide accessibility and anonymity, but also operate without sufficient accountability, creating an environment in which individuals must navigate risks independently. This indicates that online sex work is not simply enabled by technology, but is produced through the interaction between restrictive legal frameworks, digital infrastructures, and socio-economic inequalities.

Synthetically, this research positions online sex work through MiChat as a structurally embedded and socially negotiated practice sustained by micro-level legitimacy and institutional limitations. While it provides short-term economic relief, it simultaneously reproduces multiple forms of vulnerability across psychological, social, and symbolic dimensions. Therefore, policy responses must move beyond moral regulation and instead address the structural drivers of participation. This includes expanding access to inclusive employment opportunities, strengthening integrated health and psychosocial services, and developing more accountable governance frameworks for digital platforms. Ultimately, an effective social welfare approach must recognize individuals not as sources of the problem, but as actors navigating constrained choices within unequal systems.

Gender inequality constitutes a central structural dimension shaping both participation and vulnerability in online sex work mediated through MiChat in Jayapura. The findings indicate that women disproportionately bear the burden of economic responsibility, caregiving obligations, and social stigma, reflecting broader patterns of gendered labor inequality in informal economies. This aligns with Sanders et al. (2017), who argue that digital sex work is deeply embedded within existing gender power relations rather than operating as a neutral economic space. In the Indonesian context, these dynamics are further intensified by normative expectations surrounding femininity, morality, and sexual respectability, which position women simultaneously as economic providers and moral subjects subject to social judgment (Tawakal and Imelda, 2022). As a result, women engaged in online sex work are situated within what can be described as the feminization of vulnerability, where limited access to formal employment, unequal distribution of economic opportunities, and persistent stigma converge to constrain their choices (Lutnick, 2016). Therefore, involvement in online sex work should not be interpreted as individual deviance, but rather as a gendered survival strategy emerging from intersecting structural inequalities that disproportionately affect women in peripheral urban contexts such as Jayapura.

This study offers a novel contribution by bridging The Social Construction of Reality with a multidimensional social welfare framework, specifically through the integration of economic, social, psychological, spiritual, and health dimensions in analyzing online sex work mediated by MiChat in Jayapura. While existing studies often examine sex work either through moral discourse, legal regulation, or economic survival alone, this research advances an integrative perspective that situates individual experiences within both symbolic processes of meaning-making and structural conditions of welfare inequality. By demonstrating how the processes of externalization, objectivation, and internalization operate simultaneously across these five dimensions, this study reveals that online sex work is not only socially constructed but also deeply embedded in fragmented welfare systems that fail to provide comprehensive protection. In the specific context of Jayapura as a peripheral urban setting, this approach highlights how digital platforms intersect with localized socio-cultural norms, limited institutional capacity, and gendered economic vulnerability. Therefore, the analytical synthesis between social construction theory and multidimensional welfare analysis represents a significant theoretical and empirical contribution, offering a more holistic framework for understanding digitally mediated informal labor in contexts marked by structural inequality and limited social protection.

## 5 Conclusion

The practice of daring sex work through the MiChat app in Jayapura City cannot be understood as a single phenomenon or solely the result of individual choice, but rather as a social reality constructed within the context of structural inequality, digital technology developments, and local socio-cultural dynamics. Through a social construction perspective, the processes of externalization, objectivation, and internalization demonstrate that individuals involved do not act completely freely, but continually negotiate their involvement within conditions of economic constraints, social pressure, and weak institutional support. Research findings also indicate a differentiation of meaning based on social class position, where involvement can range from exploratory and lifestyle-oriented to a survival strategy driven by pressing economic needs and family responsibilities. Thus, daring sex work is more appropriately understood as a livelihood strategy negotiated within an unequal social structure, rather than as deviant behavior or a purely individual phenomenon.

Involvement in online sex work is closely linked to multidimensional wellbeing, encompassing economic, social, psychological, spiritual, and health dimensions. While this practice can provide short-term economic fulfillment, it also reproduces various forms of vulnerability, such as social stigma, psychological distress, moral conflict, and limited access to comprehensive health care. These vulnerabilities are not solely individual consequences, but rather result from a fragmented welfare system, weak institutional coordination, and gender inequality that disproportionately burdens women. In this context, individuals involved in online sex work are more accurately positioned as victim-survivors actively negotiating their survival, dignity, and identity in a situation of constraint, rather than as objects of moral judgment. Therefore, the persistence of this practice reflects not only individual adaptation strategies but also the failure of social protection systems to address the complexities of digital-based informal work.

This research contributes by integrating social construction theory with a multidimensional social welfare framework, enabling a more comprehensive analysis of online sex work in a peripheral urban context like Jayapura. This integration demonstrates how the process of social meaning-making intersects with conditions of structural inequality, while also revealing the limitations of the welfare system in responding to the realities of the evolving digital economy. From a policy perspective, the findings of this study emphasize the importance of shifting approaches from moralistic and repressive ones to more inclusive and structurally based interventions, such as expanding access to decent work, strengthening health services integrated with psychosocial support, and developing gender-sensitive economic empowerment programs. Furthermore, more accountable digital platform governance and stronger cross-sectoral coordination are needed to address the structural roots of vulnerability. Therefore, effective policies must position the individuals involved not as the source of the problem, but as actors attempting to survive within an unequal and minimally protected social system.

This study has several limitations that should be considered when interpreting the findings. First, the relatively limited number of informants and the in-depth qualitative approach mean that the results are not intended to be broadly generalized, but rather to provide a rich, contextual understanding of the dynamics of online sex work in Jayapura City. Second, this study focuses on the experiences of individuals involved in a specific platform (MiChat), thus not fully capturing variations in similar practices on other digital platforms or in different regional contexts. Third, limited data related to the perspectives of other actors, such as service users, law enforcement officials, and healthcare providers, also limits a more comprehensive understanding of the broader ecosystem. Therefore, future research is recommended to expand the number and diversity of informants, use a comparative approach across regions or platforms, and integrate multi-actor perspectives to produce a more holistic analysis. Furthermore, future studies could also explore in more depth the relationship between digital regulation, social policy, and gender inequality in shaping technology-based informal work practices in Indonesia.

## Data Availability

The original contributions presented in the study are included in the article/supplementary material, further inquiries can be directed to the corresponding author.

## References

[B1] Abidjulu F. C. (2019). Persepsi Publik Terhadap Kebijakan Penutupan Lokalisasi Prostitusi Tanjung Elmo di Kabupaten Jayapura. J. Pemberdayaan Masy. Papua 1, 17–23.

[B2] Adli W. Cangara H. Wahid U. (2023). Analisis Komunikasi Pada Aplikasi Michat Sebagai Sarana Media Prostitusi Online Di Ibu Kota Jakarta. Innov. J. Soc. Sci. Res. 3, 1834–1848.

[B3] Awaludin A. (2019). “The uncertainty of regulating online prostitution in Indonesia,” in 3rd International Conference on Globalization of Law and Local Wisdom (ICGLOW 2019) (Surakarta: Atlantis Press), 332–334. doi: 10.2991/icglow-19.2019.81

[B4] Awaludin A. Triana I. (2019). “Moral panic and online prostitution: study of social reactions,” in First International Conference on Progressive Civil Society (ICONPROCS 2019) (Yogyakarta: Atlantis Press), 277–280. doi: 10.2991/iconprocs-19.2019.58

[B5] Berger P. L. Luckmann T. (1966). The Social Construction of Reality: A Treatise in the Sociology of Knowledge. New York, NY: Anchor Books.

[B6] Bernstein E. (2007). Temporarily Yours: Intimacy, Authenticity, and the Commerce of Sex. Chicago, IL; University of Chicago Press. doi: 10.7208/chicago/9780226044620.001.0001

[B7] Buloto A. V. (2024). Anomitas MiChat dan Implikasi Hukum: Diskursus Kajian Penyalahgunaan Aplikasi MiChat Sebagai Wadah Prostitusi Online Berdasarkan Pasal 27 Ayat 1 UU No. 19 Tahun 2016. J. Cendekia Ilm. 4, 852–861.

[B8] Creswell J. W. Poth C. N. (2016). Qualitative Inquiry and Research Design: Choosing among Five Approaches. Thousand Oaks, CA: Sage publications.

[B9] Farley M. Baral I. Kiremire M. Sezgin U. (1998). Prostitution in five countries: violence and post-traumatic stress disorder. Fem. Psychol. 8, 405–426. doi: 10.1177/0959353598084002

[B10] Goehring G. (2025). Fallen women: recessions and the supply of sex work. J. Public Econ. 247:105405. doi: 10.1016/j.jpubeco.2025.105405

[B11] Goffman E. (1963). Stigma: Notes on the Management of Spoiled Identity. Englewood Cliffs, NJ: Prentice-Hall.

[B12] Habib M. A. F. Ramadhani M. Fatkhullah M. (2024). Strategi digital marketing atas produk dan layanan yang ditawarkan dalam bisnis prostitusi online. J. Iptekkom 26, 155–168.

[B13] Hamid F. Djamali D. Ahriani A. (2024). Fenomena Prostitusi Dengan Menggunakan Aplikasi MiChat Di Kota Sorong. Al-Hikmah J. Dakwah Komun. 3, 73–82. doi: 10.47945/al-hikmah.v3i2.1561

[B14] Islam A. I. Hartini S. Purwaningsih P. (2024). Law enforcement analysis against online prostitution brokers through the michat application: a study at the cibinong district court. J. Mhs. Yustisi 2, 70–73. doi: 10.32832/jurmayustisi.v2i2.1199

[B15] Juditha C. (2022). Mapping the promotion of commercial sex services using the michat application: from prevention to solutions. Profetik J. Komun. 15, 256–271. doi: 10.14421/pjk.v15i2.2560

[B16] Lukas K. Umaternate A. R. (2023). Prostitusi Online Di Kota Bitung Menggunakan Michat (Sebuah Studi Fenomenologi). Discourse Indones. J. Soc. Stud. Educ. 1, 17–25. doi: 10.69875/djosse.v1i1.1

[B17] Lutnick A. (2016). Domestic Minor Sex Trafficking: Beyond Victims and Villains. New York, NY: Columbia University Press. doi: 10.7312/lutn16920

[B18] Markey P. M. Markey C. N. (2013). Seasonal variation in internet keyword searches: a proxy assessment of sex mating behaviors. Arch. Sex. Behav. 42, 515–521. doi: 10.1007/s10508-012-9996-5 22810997

[B19] Marmot M. (2005). The Status Syndrome: How Social Standing Affects Our Health and Longevity. London: Bloomsbury.

[B20] Maulidani R. Y. Suminar D. R. (2024) *Kesejahteraan Psikologis Wanita Menikah Penyedia Jasa Prostitusi Online Open Bo di Surabaya*. Surabaya: Repository Universitas Airlangga.

[B21] Miles M. B. Huberman M. (1992). Analisis Data Kualitatif . Jakarta: Penerbit, Universitas Indonesia.

[B22] Mudjiyanto B. Lusianawati H. Launa L. Azizah N. (2024). Media sosial dan prostitusi online (Studi penggunaan media sosial sebagai sarana amplifikasi prostitusi online). Source J. Ilmu Komun. 6, 20–36.

[B23] Natalis A. Djohan N. H. (2025). Cybersex trafficking: legal challenges and protection for women and children in Indonesia. Int. Cybersecur. Law Rev. 6, 421–456. doi: 10.1365/s43439-025-00149-1

[B24] Nicolas V. R. Maharani A. E. P. (2024). “Reformulating the legal policy for online prostitutes based on electronic information and transactions law viewed from legal certainty,” in International Conference on Cultural Policy and Sustainable Development (ICPSD 2024) (Surakarta: Atlantis Press), 585–589. doi: 10.2991/978-2-38476-315-3_79

[B25] Pargament K. I. (2001). The Psychology of Religion and Coping: Theory, Research, Practice. New York, NY: Guilford Press.

[B26] Perawati A. Dompak T. (2024). Peran Aplikasi Kencan Online dalam Fasilitas Perdagangan Seksual di Indonesia. J. Adm. Negara. 1, 1–9.

[B27] Rahawarin D. Y. F. (2025). Analisis cyber crime tentang prostitusi online. Mansinam Law Rev. 2, 68–79.

[B28] Sanders T. Scoular J. Campbell R. Pitcher J. Cunningham S. (2017). Internet Sex Work: Beyond the Gaze. Cham: Springer. doi: 10.1007/978-3-319-65630-4

[B29] Sari R. P. N. Idris S. Sumby E. B. Putri M. (2025). Religiosity conflict and social reality in the practice of prostitution: a study on female sex workers in karang dempel. Musabab J. Islam. Law Soc. Pract. 1, 78–91.

[B30] Smith M. Mac J. (2018). Revolting Prostitutes: The Fight for Sex Workers' Rights. London: Verso Books.

[B31] Sonjaya S. (2025). Mengeksplorasi Regulasi Industri Seksual di Indonesia: Pemidanaan Pembeli Jasa Pekerja Seks Komersial. JCIC J. CIC Lemb. Riset Konsultan Sos. 7, 23–38. doi: 10.51486/jbo.v7i1.221

[B32] Sukardi E. Pasaribu D. Jennifer G. Kaliye V. X. (2021). Memberantas Prostitusi Online Pada Masa Pandemi Covid-19 Melalui Sosialisasi Hukum Perspektif Teori Keadilan Bermartabat. J. Lemhannas RI 9, 99–113. doi: 10.55960/jlri.v9i1.380

[B33] Tawakal M. I. Imelda J. D. (2022). Transformation of prostitution services and stigma against prostitutes (WTS): literature review. Soshum J. Sos. Hum. 12, 117–131. doi: 10.31940/soshum.v12i2.117-131

[B34] Thoits P. A. (2011). Mechanisms linking social ties and support to physical and mental health. J. Health Soc. Behav. 52, 145–161. doi: 10.1177/0022146510395592 21673143

[B35] Yunus J. O. Mahendra I. G. A. A. (2020). Pengembangan Strategi Media Sosial dalam Penjangkauan Pekerja Seks Perempuan untuk Tes HIV Mandiri. Available online at: https://www.researchgate.net/publication/348959994 (Accessed March 20, 2026).

[B36] Zelizer V. A. (2005). The Purchase of Intimacy. Princeton, NJ: Princeton University Press.

